# Juglone and KPT6566 Suppress the Tumorigenic Potential of CD44^+^CD133^+^ Tumor-Initiating Caco-2 Cells *In Vitro* and *In Vivo*

**DOI:** 10.3389/fcell.2022.861045

**Published:** 2022-03-30

**Authors:** Junghoon Kim, Seonock Lee, Ruijing Sun, Jungho Kim

**Affiliations:** Laboratory of Molecular and Cellular Biology, Department of Life Science, Sogang University, Seoul, South Korea

**Keywords:** Juglone, KPT6566, Pin1 inhibitor, Caco-2, tumor-initiating cells

## Abstract

Pin1, a *cis*/*trans* isomerase of peptidyl-prolyl peptide bonds, plays a crucial role in the pathogenesis of many human cancers. Although chemical inhibitors of Pin1 show potent antitumor therapeutic properties against various cancers, their effect on colorectal cancer, especially colorectal tumor-initiating cells, remains unknown. Here, we investigated the effect of Juglone and KPT6566 on Caco-2 cells and tumor-initiating Caco-2 cells. Juglone and KPT6566 inhibited cell growth and colony formation, and induced apoptosis of Caco-2 cells. We also found that Juglone and KPT6566 downregulated expression of G1-phase-specific cyclins and cyclin-dependent kinases in a time-dependent manner, consistent with suppression of Caco-2 cell proliferation and colony formation. Although tumor-initiating cells are thought to be responsible for resistance to traditional chemotherapeutic drugs, our experiments demonstrate that Juglone or KPT6566 kill both tumor-initiating and non-tumor-initiating Caco-2 cells with equal or similar efficacy. Finally, when CD44^+^CD133^+^ tumor-initiating Caco-2 cells were injected into NSG mice, Juglone or KPT6566 led to a meaningful reduction in tumor volume and mass compared with tumors isolated from mice that received control treatment. Overall, these results indicate that chemical Pin1 inhibitors may be a valuable therapeutic option against colorectal tumor-initiating cancer cells.

## Introduction

Human colorectal cancer (CRC; also referred to as bowel cancer, rectal cancer, or colon cancer) is one of the most prevalent causes of cancer mortality among men and women worldwide (Siegel R. L. et al., 2020; Siegel R. L. et al., 2020). It originates from adenomatous polyps, which are non-malignant precursor lesions that are not pre-cancerous; however, they can sometimes change into malignant cancers through sequential genetic and epigenetic alterations (Markowitz and Bertagnolli, 2009). Although clinical studies conducted worldwide have tried many novel strategic treatments to overcome the limitations of conventional cancer treatment, CRC remains difficult to cure, and many cases are fatal (Meyerhardt and Mayer, 2005). Thus, a better understanding of the molecular pathways contributing to colorectal carcinogenesis is necessary to develop a new strategy for chemotherapy to treat human CRC.

Drug resistance is a significant limitation of traditional chemotherapeutic drugs, including those used to treat CRC (Tredan et al., 2007; Van Der Jeught et al., 2018). Although successful chemotherapy abolishes the bulk of the cancer, only non-tumor-initiating cells (non-cancer stem cells) are eliminated. Recent reports suggest that a subpopulation of cancerous cells (called tumor-initiating cells or cancer stem cells) is responsible for cancer drug resistance and recurrence (Dean et al., 2005; Visvader and Lindeman, 2008; Alison et al., 2011; Li et al., 2021). Because ABCG2 and other drug transporter proteins are often upregulated in tumor-initiating cells, these cells are less sensitive to most conventional chemotherapeutic drugs than non-tumor-initiating cells. Eventually, tumor-initiating cells are enriched in human cancer tissues after chemotherapy (Hirschmann-Jax et al., 2004; Dean et al., 2005; Seigel et al., 2005; Haraguchi et al., 2006; Visvader and Lindeman, 2008; Alison et al., 2011). Currently, colorectal tumor-initiating cells are a challenging therapeutic target, but successful therapy could resolve the chemotherapeutic difficulties associated with human CRC. Thus, new approaches to targeting colorectal tumor-initiating cells are needed desperately to improve prognoses.

*Cis-trans* isomerization of proteins leads to post-translational structural changes, a process essential for cancer development (Lu et al., 2007; Makinwa et al., 2020). Isomerization between the *cis* and *trans* conformations of peptide bonds is catalyzed by PPIases (peptidyl-prolyl *cis*/*trans* isomerases). Interestingly, Pin1 (peptidyl-prolyl *cis*/*trans* isomerase or NIMA-interacting 1) is the only identified PPIase that can recognize and isomerize the phosphorylated serine-proline (pSer-Pro) or phosphorylated threonine-proline (pThr-Pro) sequences of target proteins (Lu and Zhou, 2007). In human cancers, Pin1-mediated prolyl *cis/trans* isomerization induces a conformational change in its substrates and regulates the stability of its target proteins, which are involved in tumorigenesis (Yu et al., 2020). Additionally, Pin1 is overexpressed in most human cancers, and elevated Pin1 expression promotes cancer formation and is positively associated with an unfavorable prognosis (Wulf et al., 2001; Wulf et al., 2004; Lu and Hunter, 2014; Luo et al., 2014; Rustighi et al., 2014); by contrast, lack of Pin1 hampers tumor development in mouse models (Wulf et al., 2004; Girardini et al., 2011; D’artista et al., 2016). In addition, several chemical Pin1 inhibitors, including Juglone, KPT-6566, and all-*trans*-retinoic acid, have antitumor effects, indicating the critical role of Pin1 in tumorigenesis (Kim et al., 2009; Wei et al., 2015; Campaner et al., 2017). Juglone, or 5-hydroxy-1,4-naphthoquinone, was isolated initially from black walnut skin (Hennig et al., 1998). Biochemical experiments show that Juglone irreversibly inhibits the PPIase activity of PIN1 (Hennig et al., 1998). More recently, studies show that Juglone inhibits proliferation of human leukemias, breast, and gastric cancers (Namgoong et al., 2010; Ji et al., 2011; Xu et al., 2012; Nakatsu et al., 2020). KPT6566 {2-[(4-(4-*tert*-butylbenzenesulfonamido)-1-oxo-1,4-dihydronaphthalen-2-yl)sulfanyl]acetic acid} is another potent inhibitor identified from Pin1 inhibitor screening of a commercial library (Campaner et al., 2017). KPT6566 binds covalently to the catalytic domain of Pin1 and selectively inhibits Pin1 catalytic activity; however, Juglone is likely to inhibit targets other than Pin1 (Chao et al., 2001; Campaner et al., 2017; Ahmad and Suzuki, 2019). Interestingly, KPT6566 can induce Pin1 degradation in an *ex vivo* system (Campaner et al., 2017).

Even though overexpression of Pin1 is implicated in various tumors, little is known about its effect on CRC or colorectal tumor-initiating cells. In the present study, we asked whether Juglone or KPT6566 inhibit the viability and colony forming capability of Caco-2 cells to suppress the tumorigenic potential of CD44^+^CD133^+^ tumor-initiating Caco-2 cells. We show that Juglone and KPT6566 suppress proliferation and colony formation of CD44^+^CD133^+^ tumor-initiating Caco-2 cells *in vitro*, and hamper the tumorigenic capacity of CD44^+^CD133^+^ tumor-initiating Caco-2 cells *in vivo*. The results suggest that targeting Pin1 in colorectal tumor-initiating cells is an appealing therapeutic approach to human CRC. In particular, targeting tumor-initiating cells using chemical Pin1 inhibitors provides a new option for management of CRC tumor-initiating cells.

## Materials and Methods

### Cell Culture

Human colorectal cancer cell lines (Caco-2, HCT116, HT29, SW480, and DLD-1) were obtained from the American Type Culture Collection and grown at 37°C in EMEM (Eagle’s Minimum Essential Medium) supplemented with 10% heat-inactivated FBS (fetal bovine serum; Sigma-Aldrich), GlutaMAX (ThermoFisher Scientific), and 1% penicillin-streptomycin (ThermoFisher Scientific) in a humidified cell culture incubator (ThermoFisher Scientific) with 5% CO_2_.

### Cell Growth Curve

Caco-2 cells (2 × 10^4^) were plated in duplicate in 12-well plates and cultured for 6 days. At 2 days intervals, cells were observed under an inverted phase-contrast microscope (IX71; Olympus). Total cell numbers were counted at 2 days intervals over a period of 6 days using a hemocytometer. The average value was recorded.

### Western Blot Analysis

Cell extracts were separated by SDS-PAGE. The lysate proteins were transferred from the gel to a PVDF membrane and immunoblotted with anti-Pin1 (10495-1-AP, Proteintech), anti-cyclin D1 (MAI-39546; Invitrogen), anti-cyclin D2 (#3741; Cell Signaling Technology), anti-cyclin D3 (#2936; Cell Signaling Technology), anti-CDK4 (SC-23896; Santa Cruz Biotechnology), anti-CDK6 (SC-53638; Santa Cruz Biotechnology), anti-β-catenin (610154; BD Transduction Laboratories), anti-β-actin (AbC-2002, AbClon), or anti-α/β-tubulin (#2148S; Cell Signaling Technology) antibodies. Detection was performed by chemiluminescence using Western Lightning reagent (PerkinElmer Life Sciences).

### Colony Formation Assay

For the colony formation assay, 2.5 × 10^3^ Caco-2, HCT116, HT29, SW480, or DLD-1 cells were plated in 12-well culture dishes and incubated with different concentrations of Juglone (Sigma-Aldrich) or KPT6566 (CSNpharm). After 5 days, colonies were stained with fix/staining solution [0.05% (w/v) Crystal Violet (Sigma-Aldrich), 1% formaldehyde (Sigma-Aldrich), 1% methanol (Sigma-Aldrich), and 1 × PBS] for 20 min. Colonies were counted using the ImageJ program (https://imagej.nih.gov/ij/) to quantify the reduction in CRC cell colony formation after Juglone or KPT6566 treatment. The number of colonies was compared with that in the control group.

### Cell Viability Assay to Determine IC_50_ Values

Caco-2, HCT116, HT29, SW480, DLD-1, CD44^+^CD133^+^ tumor-initiating Caco-2, or ∆CD44^+^CD133^+^ non-tumor-initiating Caco-2 cells (1 × 10^4^) were plated in each well of 12-well plates, and Juglone or KPT6566 was added to the cell culture medium at different concentrations (0, 0.625, 1.25, 2.5, 5, or 10 μM Juglone; 0, 1.25, 2.5, 5, 10, or 20 μM KPT6566). Cell viability after treatment was measured using the ADAM MC Auto Cell Counter System (NanoEntek). The half-maximal inhibitory concentration (IC_50_) of Juglone and KPT6566 was determined using the SoftMax Pro software (Molecular Devices).

### Cell Cycle Analysis

Following treatment with Juglone or KPT6566, Caco-2 cells were fixed overnight at 4°C with fixation solution (70% ethanol), washed twice in 1 × PBS, resuspended in RNase solution (100 μg/ml RNase A in 1 × PBS), and incubated at 37°C for 30 min. The fixed Caco-2 cells were then stained with propidium iodide (PI) solution (33 μg/ml PI containing 10% NP-40) for 30 min. A histogram showing DNA content was obtained by flow cytometry (FACSCalibur; BD Biosciences).

### Apoptosis Assay

Apoptosis assays were carried out by flow cytometry using a fluorescein isothiocyanate (FITC) Annexin V Apoptosis Detection Kit with PI (BioLegend). After treatment with Juglone or KPT6566, Caco-2 cells were washed twice with Cell Staining Buffer (BioLegend) and resuspended in Annexin V Binding Buffer, according to the manufacturer’s instructions. Caco-2 cells were stained with FITC-conjugated Annexin V and PI, and then analyzed with a FACSCalibur flow cytometer.

### Fluorescence-Activated Cell Sorting

Anti-human CD44 (mouse, fluorescein-5-isothiocyanate (FITC)-conjugated; G44-26, BD Biosciences) and anti-human CD133 [mouse, phycoerythrin (PE)-conjugated; AC133, Miltenyi Biotech] antibodies were used for standard cell surface flow cytometry. Caco-2 cells were sorted using a FACSVantage SE flow cytometer (BD Biosciences) after staining with FITC-conjugated anti-CD44 and PE-conjugated anti-CD133 antibodies. Flow cytometry data were analyzed by CellQuest software (BD Biosciences).

### Reverse Transcription-PCR

TRIzol solution (Invitrogen) was used to isolate total RNAs from CD44^+^CD133^+^ and ∆CD44^+^CD133^+^ Caco-2 cells. Next, cDNAs were synthesized using a Superscript First-strand Synthesis System (Invitrogen), and RT-PCR reactions to amplify *Pin1* were performed in triplicate. β-actin mRNA was used as an internal control. The following primers were used: Pin1: 5′-TCA​GGC​CGA​GTG​TAC​TAC-3′ and 5′-CGG​AGG​ATG​ATG​TGG​ATG-3′ (https://www.ncbi.nlm.nih.gov/nuccore/NM_006221.4) (Yang et al., 2013); and β-actin: 5′-GCT​CGT​CGT​CGA​CAA​CGG​CTC-3′ and 5′-CAA​ACA​TGA​TCT​GGG​TCA​TCT​TCT​C-3′ (https://www.ncbi.nlm.nih.gov/nuccore/NM_001101.5).

### Quantitative Real-Time PCR

Quantitative real-time PCR was performed on a LightCycler 96 Real-Time PCR System (Roche) using BioFACT 2X Real-Time PCR Kit (With SFCgreen I) (BioFACT), according to the manufacturer’s instructions. Reaction conditions for optimal amplification of Pin1 were determined as described in the manufacturer’s manual. As an internal control, expression of mRNA encoding β-actin was also quantified. Relative expression of Pin1 was calculated and normalized to that of β-actin using the ∆C_T_ method (Schmittgen and Livak, 2008).

### Measurement of Tumor Volume

To measure tumor suppression by Juglone or KPT6566, CD44^+^CD133^+^ tumor-initiating Caco-2 cells were resuspended in PBS (1:1 ratio of PBS and Matrigel), and 1 × 10^5^ cells were injected into the left and right flanks of 8 week-old NSG mice using a 21-gauge needle. When tumors reached 15–25 mm^3^, 5 mg/kg Juglone or KPT6566 per NSG mouse was injected intraperitoneally once every 3 days for 30 days. An equivalent volume of vehicle was used as the control. All mice that developed tumors were monitored carefully and sacrificed 30 days after treatment started. Mice were weighed at 3 days intervals using a digital scale, and the tumor length and width were measured at 2 days intervals using a digital external caliper. Tumor volume was calculated using the following formula (Euhus et al., 1986; Tomayko and Reynolds, 1989; Kim et al., 2019):

Tumor volume = (L × W^2^)/2, where L = length (the greatest longitudinal diameter) and W = width (the greatest transverse diameter).

All experiments were carried out in accordance with the animal experimentation guidelines at Sogang University, and all mouse experiments were approved by the institutional committee (IACUCSGU 2020-18).

### Statistical Analysis

All experimental data are presented as the mean ± S.D. (standard deviation). An unpaired Student’s t-test was used to compare the means between two experimental groups. Statistical analysis was performed using Microsoft Excel (Microsoft Corporation). A *p* value < 0.05 was deemed statistically significant.

## Results

### Juglone and KPT6566 Inhibit the Colony Formation Ability of CRC Cell Lines

**FIGURE 1:**
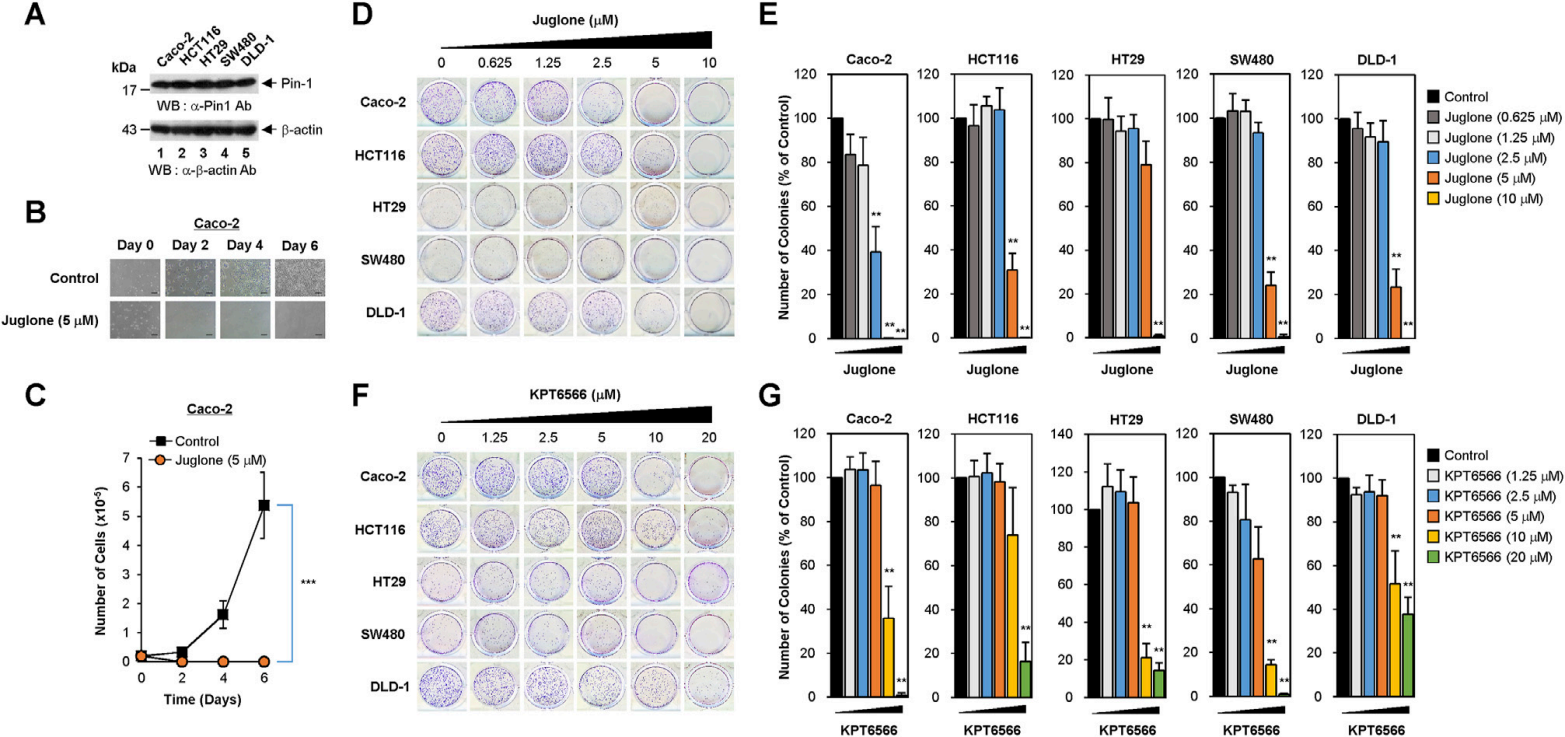
Dose-dependent inhibition of human colorectal cancer cell colony formation by Juglone or KPT6566. **(A)** Expression of Pin1 by colorectal cancer cell lines. Caco-2 (lane 1), HCT116 (lane 2), HT29 (lane 3), SW480 (lane 4), or DLD-1 (lane 5) human colorectal cancer cells were harvested and cell lysates prepared. Total protein was fractionated by SDS-PAGE (15% gels for anti-Pin1 and 12% gels for anti-β-actin blots), transferred to a PVDF membrane, and immunoblotted with anti-Pin1 (upper panel) or β-actin (bottom panel) antibodies as indicated. The position of the prestained protein molecular weight marker (New England Biolabs) is indicated to the left of the gel (kDa). Similar results were obtained from three independent experiments. **(B)** Morphology of Caco-2 cells exposed to Juglone. Caco-2 cells (2 × 10^4^) were plated into 12-well culture plates and incubated with Juglone (5 μM). Cells were monitored at 2 days intervals for 6 days under an inverted phase-contrast microscope (IX71; Olympus). Scale bars, 100 μm. Similar results were obtained from three independent experiments. **(C)** Inhibition of Caco-2 cell growth by Juglone. Caco-2 cells were seeded at 2 × 10^4^ cells/well and grown in EMEM supplemented with or without 5 μM Juglone. Cells were counted at 2 days intervals for a total of 6 days using a hemocytometer. Values are expressed as the mean ± S.D. Similar results were obtained from three independent experiments. Statistical significance was determined using an unpaired Student’s t-test. ***p < 0.001, versus control group. **(D)** Suppression of colony formation by Juglone. Caco-2 (top panels), HCT116 (second panels), HT29 (third panels), SW480 (fourth panels), and DLD-1 cells (bottom panels) were seeded at a density of 2.5 × 10^3^ per well and cultured in medium containing 0, 0.625, 1.25, 2.5, 5, or 10 μM Juglone. Colony formation assays were performed 5 days later, and colonies were visualized by staining with 0.05% (w/v) of Crystal Violet. Representative images are shown. Three independent experiments yielded similar results. **(E)** Quantification of the reduction in Caco-2 (first graph), HCT116 (second graph), HT29 (third graph), SW480 (fourth graph), and DLD-1 cells (fifth graph) colony formation after Juglone treatment. Colonies were counted after Crystal Violet staining, and the number of colonies formed was compared with that in the untreated group (0 μM Juglone). Similar results were obtained from three independent experiments. The value in the control group was normalized to 100. Values represent the mean ± S.D. Statistical significance was determined using an unpaired Student’s t-test. **p < 0.01, versus control cells. **(F)** Inhibition of colony formation by KPT6566. The same human colorectal cancer cells were seeded at a density of 2.5 × 10^3^ per well and cultured in medium containing 0, 1.25, 2.5, 5, 10, or 20 μM KPT6566. Colony formation assays were performed 5 days later, and colonies were visualized by staining with 0.05% (w/v) Crystal Violet. Representative images are shown. Similar results were obtained in three independent experiments. **(G)** Relative colony number after KPT6566 treatment. To quantify the reduction in colorectal cancer cell colony formation after KPT6566 treatment, Caco-2, HCT116, HT29, SW480, and DLD-1 cells were visualized by staining with 0.05% (w/v) Crystal Violet. Independent experiments were repeated at least three times, and triplicate wells (at least) were used. Values are expressed as the mean ± S.D., and statistical analysis was done using an unpaired Student’s t-test. **p < 0.01, versus control cells.

Overexpression of Pin1 has been reported in many human cancers and cancer cell lines (Lu, 2003). Also, Pin1 is overexpressed in human CRC patients (Kim et al., 2005; Kuramochi et al., 2006). However, little is known about whether targeting Pin1 suppresses the tumorigenic potential of human CRC. To investigate whether inhibition of Pin1 prevents CRC cell growth, we performed western blot analysis to measure expression of Pin1 in five representative colorectal cancer cell lines, namely, Caco-2, HCT116, HT29, SW480, and DLD-1. We detected high levels of Pin1 protein in all five human CRC cell lines (Figure 1A). Also, we tried immunocytochemistry analysis to assess the expression pattern of Pin1 in Caco-2 cells. The results showed that Pin1 protein was present in Caco-2 cells. Interestingly, the expression pattern of Pin1 in individual Caco-2 cells was not homogeneous. As shown in Supplementary Figure S1A, cell-level heterogeneity of Pin1 expression was observed in Caco-2 cells in each sampled region of a slide. We also performed immunocytochemistry experiments using the HCT116, HT29, SW480, and DLD-1 cell lines, and observed similar results (Supplementary Figures S1B–E).

To assess the potential antiproliferative effects of chemical Pin1 inhibitors in CRC, 2 × 10^4^ Caco-2 cells were seeded into 12-well plates and treated with 5 μM of Juglone (a chemical Pin1 inhibitor) for the indicated times. Cells were monitored for 6 days. Control Caco-2 cells showed epithelial-like cell morphology and formed colonies; however, 5 μM Juglone caused a time-dependent decrease in Caco-2 cell growth (Figure 1B).

To quantify suppression of proliferation by Juglone, the number of Caco-2 cells was counted at 2 days intervals for 6 days. Juglone treatment caused significant suppression of Caco-2 cell proliferation compared with control cells (Figure 1C).

Since the colony forming capability of cancer cells is used to gauge the transforming ability of cells responsible for tumorigenic potential, we wanted to test whether Juglone inhibits the clonal growth of CRC cells. To investigate whether Juglone affects colony formation by Caco-2 cells, 2.5 × 10^3^ cells were plated and treated with the indicated concentrations of Juglone (0–10 μM), and colonies were visualized 5 days later by Crystal Violet staining. Colony formation assays showed that colon cancer cells treated with different concentrations of Juglone were less able to form colonies than control colon cancer cells. As shown in Figure 1D, Juglone induced a dose-dependent decrease in the colony forming ability of Caco-2 cells (top panels). We also investigated whether Juglone suppresses the colony forming potential of other colorectal cancer cells. HCT116 (second panels), HT29 (third panels), SW480 (fourth panels), and DLD-1 cells (bottom panels) were treated with different doses of Juglone, and final measurements were taken 5 days later. Similar to Caco-2 cells, Juglone also decreased the colony forming ability of HCT116, HT29, SW480, and DLD-1 cells in a dose-dependent manner (control cells = 0 μM Juglone).

To determine the number of colonies, images of cell culture plates were analyzed using the ImageJ program after Crystal Violet staining. Treatment with 2.5, 5, or 10 μM Juglone decreased the number of Caco-2 cell colonies by 60, 99, and ∼100%, respectively (Figure 1E, first graph). While treatment with 2.5 μM Juglone had a marginal effect on the colony forming ability in HCT116 cells, 5 μM or 10 μM Juglone reduced colony formation by ∼70 and 99%, respectively (Figure 1E, second graph). We performed the same experiments using HT29, SW480, and DLD-1 colorectal cancer cell lines to further validate this observation. Treatment with 2.5, 5, or 10 μM Juglone decreased the number of HT29 colonies by 5, 20, and ∼99% (Figure 1E, third graph), the number of SW480 colonies by 7, 76, and ∼99% (Figure 1E, fourth graph), and the number of DLD-1 colonies by 10, 77, and ∼100% (Figure 1E, fifth graph), respectively.

To confirm these results, we evaluated the potential anti-colony forming effects of KPT6566. Similarly, Caco-2, HCT116, HT29, SW480, and DLD-1 cells were incubated with different concentrations of KPT6566 (0–20 μM). Colony formation assays revealed that CRC cells treated with increasing concentrations of KPT6566 were less able to form colonies than control (DMSO-treated) cells (Figure 1F). Treatment with 10 μM or 20 μM KPT6566 decreased colony formation by Caco-2 cells by ∼64 and 99% (Figure 1G, first graph), that by HCT116 cells by ∼26 and 83% (Figure 1G, second graph), that by HT29 cells by 79 and 85% (Figure 1G, third graph), that by SW480 cells by 85 and 99% (Figure 1G, fourth graph), and that by DLD1 cells by 49 and 63% (Figure 1G, fifth graph), respectively. Thus, Juglone and KPT6566 inhibit the colony forming ability of CRC cells, suggesting the potential of chemical Pin1 inhibitors to suppress tumorigenic properties. In addition, the CRC cell lines tested in this study were more resistant to KPT6566 than to Juglone.

### Juglone and KPT6566 Suppress CRC Cell Growth *ex vivo*

**FIGURE 2:**
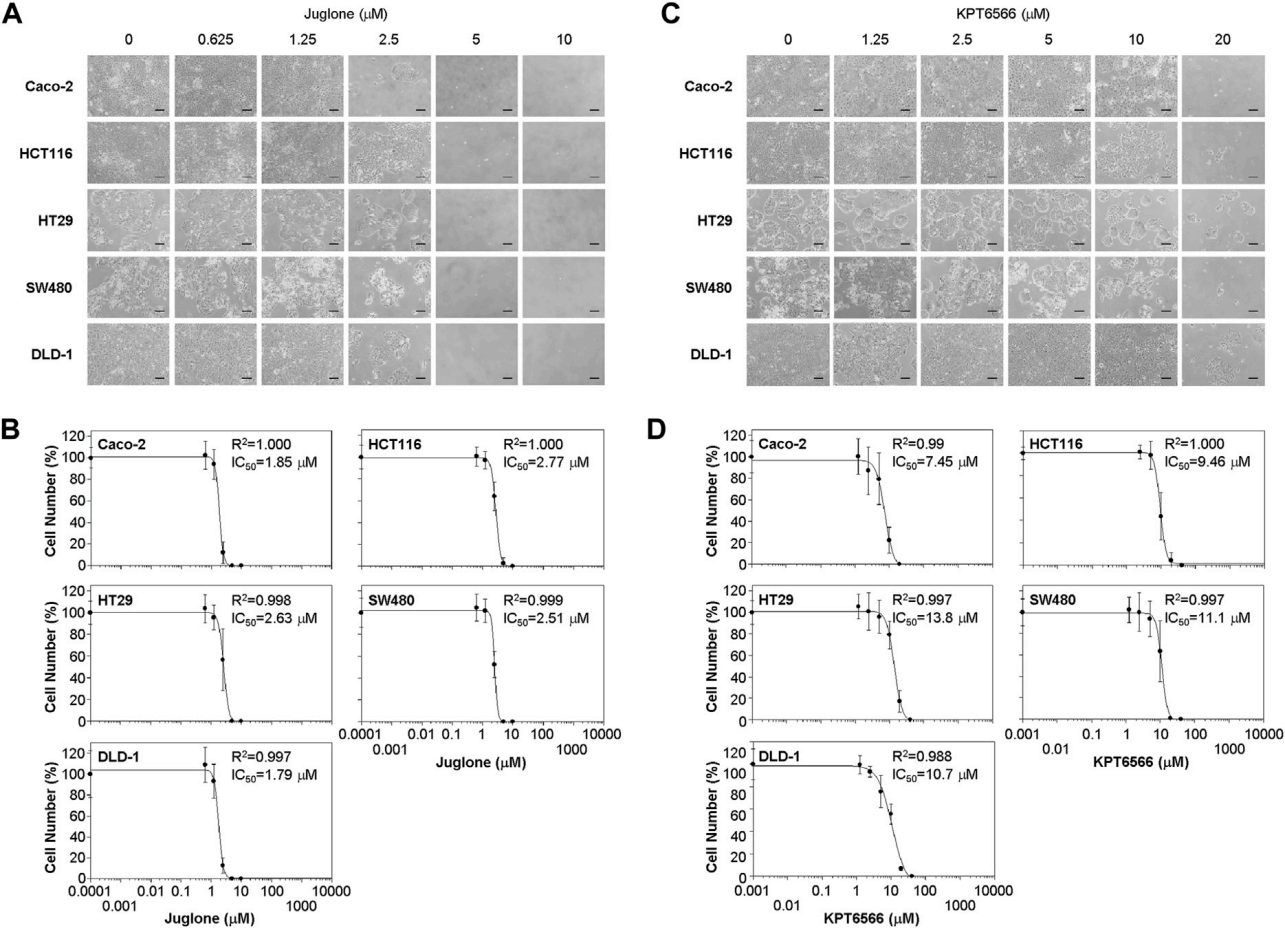
Suppression of colorectal cancer cell proliferation by Juglone and KPT6566. **(A)** Morphology of five colorectal cancer cell lines exposed to Juglone. Caco-2, HCT116, HT29, SW480, and DLD-1 cells (1 × 10^4^) were cultured with various amounts of Juglone. Phase-contrast micrographs show the growth of individual colorectal cancer cells 6 days after Juglone treatment. Individual colorectal cancer cells were monitored under an inverted phase-contrast microscope (IX71; Olympus). Scale bars, 100 μm. Three independent experiments were performed, all of which gave similar results. **(B)** Inhibition of colorectal cancer cell proliferation by Juglone. Caco-2, HCT116, HT29, SW480, and DLD-1 colorectal cancer cells were treated with the indicated concentrations of Juglone, and the number of cells was counted using a hemocytometer. Inhibition of cell growth is presented as a percentage relative to untreated control (DMSO) cells at a given concentration of Juglone. Data are expressed as the mean ± S.D. of four independent experiments. A sigmoidal dose-response curve provided by SoftMax Pro software was used to calculate the IC_50_ value for Juglone. Three independent experiments were performed, all of which gave similar results. **(C)** Effect of KPT6566 on colorectal cancer cell growth. Caco-2, HCT116, HT29, SW480, and DLD-1 cells (1 × 10^4^) were incubated with different doses of KPT6566 and monitored for 6 days under an inverted phase-contrast microscope (IX71; Olympus). Scale bars, 100 μm. Three independent experiments were performed, all of which gave similar results. **(D)** Inhibition of colorectal cancer cell proliferation by KPT6566. Caco-2, HCT116, HT29, SW480, and DLD-1 colorectal cancer cells were treated with the indicated concentrations of KPT6566, and the number of cells was counted using a hemocytometer. Inhibition of cell growth is presented as a percentage relative to untreated control (DMSO) cells at a given concentration of KPT6566. Data are expressed as the mean ± S.D. of four independently performed experiments. A sigmoidal dose-response curve provided by SoftMax Pro software was used to calculate the IC_50_ value for KPT6566. Three independent experiments were performed, all of which gave similar results.

The sensitivity of human colon cancer cell lines to Juglone was determined by calculating the 50% inhibitory concentration (IC_50_). To analyze the cytotoxic effects of Juglone in CRC cells, we investigated its effects on the viability of the five colorectal cancer cell lines. Growth of Caco-2 (Figure 2A, first panels), HCT116 (second panels), HT29 (third panels), SW480 (fourth panels), and DLD-1 cells (fifth panels) after Juglone treatment was examined by microscopy. As shown in Figure 2A, Juglone decreased the number of Caco-2, HCT116, HT29, SW480, and DLD-1 cells, and inhibited cell growth in a dose-dependent manner (Figure 2A).

Caco-2 and DLD-1 cells were more sensitive to Juglone than the other CRC cell lines. The IC_50_ of Juglone for inhibiting Caco-2 and DLD-1 cell growth was 1.85 and 1.79 μM, respectively. In addition, the IC_50_ of Juglone for HCT116, HT29, and SW480 cells was 2.77, 2.63, and 2.51 μM, respectively, which was slightly higher than that for Caco-2 and DLD-1 cells (Figure 2B).

We also analyzed the effect of KPT6566 on cell viability. The growth of Caco-2 (Figure 2C, first panels), HCT116 (second panels), HT29 (third panels), SW480 (fourth panels), and DLD-1 cells (fifth panels) after KPT6566 treatment was examined by microscopy. Addition of KPT6566 also inhibited proliferation of all CRC cells in a dose-dependent manner (Figure 2C).

In the case of KPT6566, Caco-2 and HCT116 cells were more sensitive than other CRC lines (Figure 2D). The IC_50_ of KPT6566 for inhibiting Caco-2 and HCT116 cell growth was 7.45 and 9.46 μM, respectively, whereas the IC_50_ for HT29, SW480, and DLD-1 cells was 13.8, 11.1, and 10.7 μM, respectively, which was higher than that for Caco-2 and HCT116 cells (Figure 2D). In general, the CRC cells studied here were more sensitive to Juglone than to KPT6566. Based on this initial screening result, we selected the Caco-2 cell line for further experiments designed to test the hypothesis that Pin1 inhibitors are efficacious against tumorigenesis of CRC.

### Juglone and KPT6566 Inhibit Expression of G1-phase Cell Cycle Regulatory Proteins in Caco-2 Cells

**FIGURE 3:**
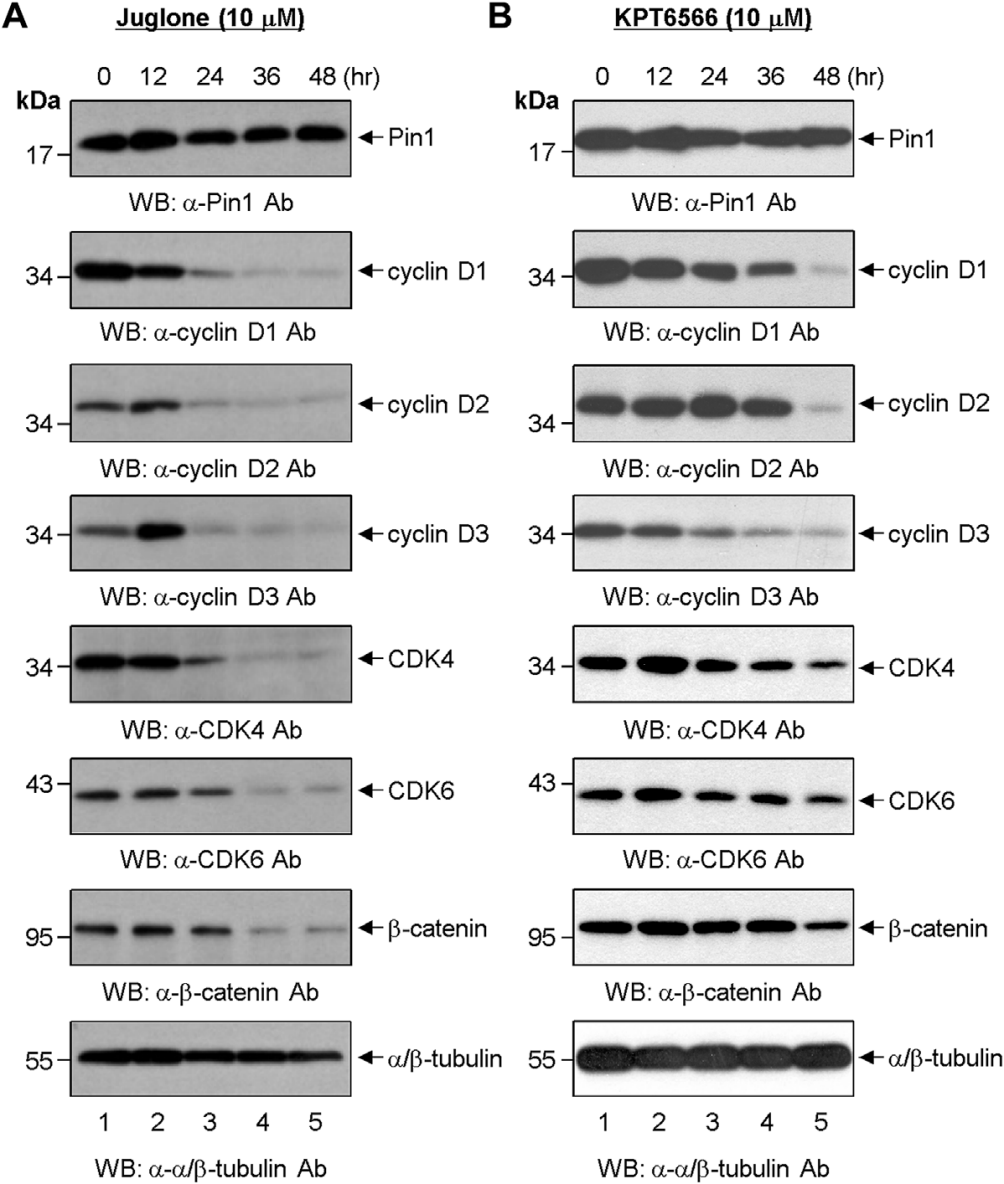
Effect of chemical Pin1 inhibitors on expression of cell cycle regulatory proteins. **(A,B)** Western blot analysis of G1-Phase cell cycle regulatory proteins after treatment with Juglone. **(A)** or KPT6566 **(B)**. Total cell extracts were prepared from Caco-2 cells at 0, 12, 24, 36, and 48 h post-treatment with Juglone or KPT6566. The cell extracts were resolved on SDS-PAGE gels (15% gels for Pin1; 12% gels for cyclin D1, cyclin D2, cyclin D3, CDK4, CDK6, and α/β-tubulin; 8% gel for β-catenin), transferred to a PVDF membrane, and immunoblotted with anti-Pin1, anti-cyclin D1, anti-cyclin D2, anti-cyclin D3, anti-CDK4, anti-CDK6, anti-β-catenin, or anti-α/β-tubulin antibodies. α/β-tubulin served as a loading control. Three independent experiments gave similar results.

Next, we investigated the effects of Juglone or KPT6566 on G1-phase cell cycle-regulating proteins in Caco-2 cells. For this, cells were treated 10 μM Juglone for 12, 24, 36, and 48 h. Juglone caused a time-dependent decrease in expression of cyclin D1, cyclin D2, cyclin D3, CDK4, and CDK6 (Figure 3A). We also found that β-catenin levels in Caco-2 cells were downregulated after treatment with Juglone. These results indicate that Juglone effectively inhibits D-type cyclins and CDK4/6 in Caco-2 cells.

The effects of KPT6566 on Caco-2 cells were also examined. We found that KPT6566 inhibited expression of D-type cyclins and CDK4/6 proteins, but appeared to be less potent than Juglone (Figure 3B). These data suggest that Juglone and KPT6566 control Caco-2 cell proliferation by downregulating expression of G1-phase cell cycle regulatory proteins. However, although KPT6566 targets Pin1 for degradation through covalent binding (Campaner et al., 2017), treatment with KPT6566 did not affect the level of Pin1 protein in Caco-2 cells significantly (Figure 3B, top panel).

### Juglone and KPT6566 Promote Apoptotic Cell Death in Caco-2 Cells

**FIGURE 4:**
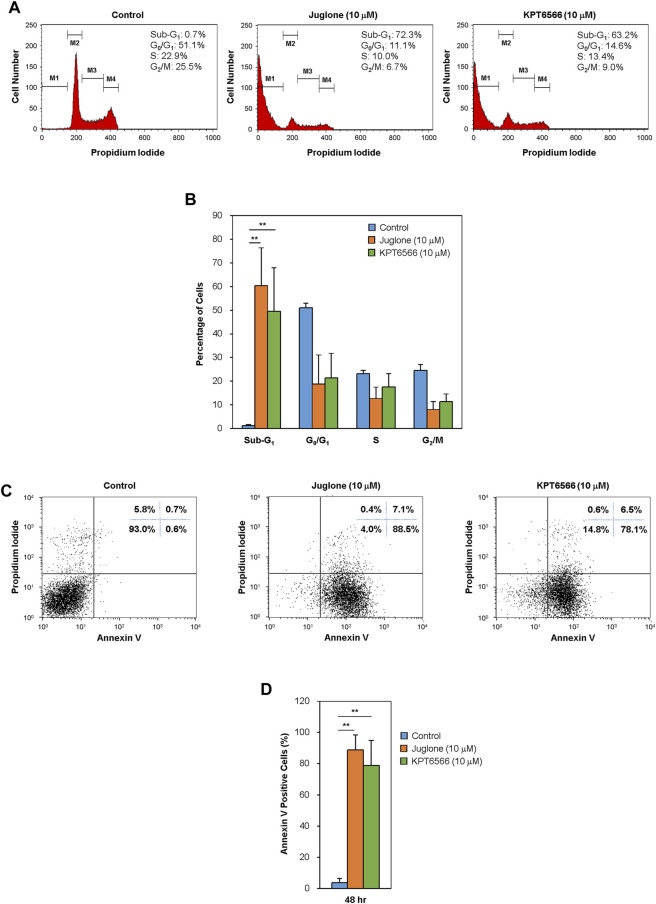
Effect of Juglone and KPT6566 on apoptosis of Caco-2 cells *in vitro*. **(A)** Flow cytometry analysis of the cell cycle distribution of Caco-2 cells after treatment with Juglone or KPT6566. Caco-2 cells were cultured for 48 h in the presence of Juglone or KPT6566, and DNA content was determined by measuring fluorescence after PI staining using a flow cytometer. M1, M2, M3, and M4 represent gated Sub-G_1_, G_0_/G_1_, S, and G_2_/M populations. Representative histograms of cell cycle distribution depict apoptosis in Juglone- or KPT6566-treated Caco-2 cells. Five independent experiments yielded similar results. **(B)** Analysis of the percentage of cells at each cell cycle phase. The percentage of total cells in sub-G_1_, G_0_/G_1_, S, and G_2_/M phase was calculated using FlowJo software. Each column represents the mean ± S.D. for each group. **p < 0.01, compared with the control group (*n* = 5). **(C)** Detection of cell apoptosis in Caco-2 cells by Annexin V-PI staining assay. Caco-2 cells were treated with 10 μM Juglone or KPT6566, stained with FITC-Annexin V-PI, and analyzed by flow cytometry. **(D)** Quantitative analysis of Juglone- or KPT6566-induced apoptosis. Apoptotic ratios for each group are presented as the mean ± S.D. **p < 0.01 versus control group (*n* = 3).

We next examined the effect of Juglone and KPT6566 on the cell cycle distribution of Caco-2 cells. Caco-2 cells treated for 48 h with 10 μM Juglone or KPT6566 were stained with PI, and the cell cycle distribution was monitored by flow cytometry. Treatment with Juglone or KPT6566 significantly increased the percentage of Caco-2 cells in the sub-G_1_ phase compared with the control group (Figure 4A). Juglone at 10 μM caused a statistically significant increase in the percentage of cells in the sub-G_1_ phase, from 0.7% (Control) to 72.3%, with a concomitant decrease in the percentage of cells in S phase, from 22.9 to 10.0% (Figure 4A, first and second panels). We also observed a marked increase in the percentage of cells in sub-G1 phase, from 0.7 to 63.2%, with a concomitant decrease in the percentage of cells in S phase, from 22.9 to 13.4%, following KPT6566 treatment (Figure 4A, first and third panels). Figure 4B shows the percentage of Caco-2 cells in sub-G_1_ phase, and percentage of cells in each phase, after treatment with Juglone (orange bars) or KPT6566 (green bars).

To explain whether the increased cell death induced by Juglone or KPT6566 was linked to apoptosis, we double-stained cells with Annexin V-FITC/PI. Annexin V binds phosphatidylserine (PS) in apoptotic cells. During apoptosis, the cell membrane changes the distribution of PS from the inner leaflet to the outer leaflet of the lipid bilayer, where it can be reached by Annexin V (Andree et al., 1990; Crowley et al., 2016). After exposure to 10 μM Juglone or KPT6566, we found a substantial increase in the percentage of apoptotic Caco-2 cells (Figure 4C). The rate of Caco-2 cell apoptosis (percentage of Annexin V-positive cells) was 88.5% after exposure to Juglone and 78.1% after exposure to KPT6566, suggesting that both Juglone and KPT6566 induce apoptosis, and that Juglone is more potent. Quantification of Annexin V-positive Caco-2 cells after exposure to Juglone or KPT6566 is shown in Figure 4D. These results strongly indicate that Juglone and KPT6566 not only reduce the number of surviving cells, but also increase the number undergoing apoptosis.

### Juglone and KPT6566 Suppress the Colony Forming Potential of CD44^+^CD133^+^ Tumor-Initiating Caco-2 Cells

**FIGURE 5:**
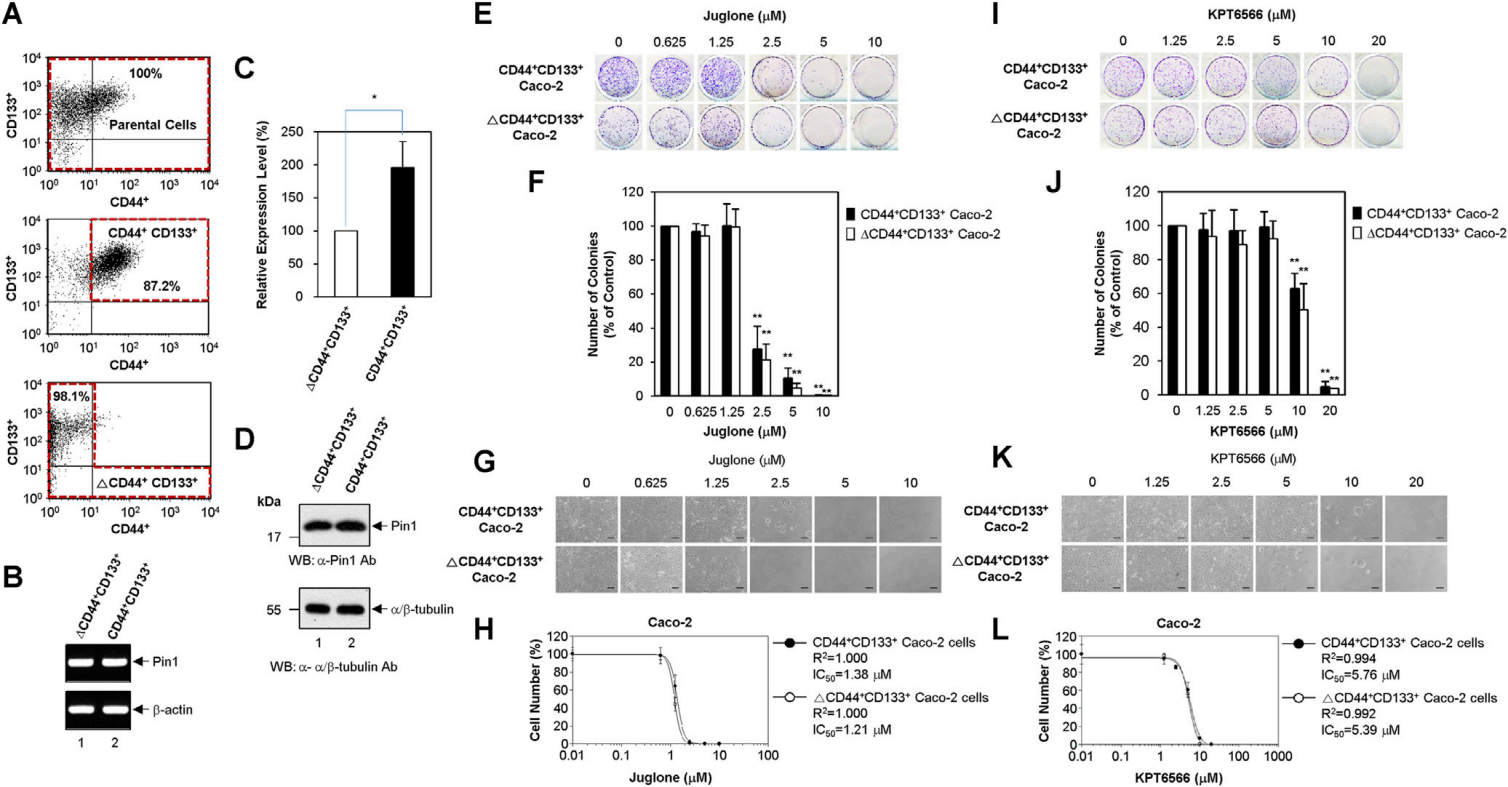
Suppression of CD44^+^CD133^+^ tumor-initiating Caco-2 cell proliferation by Juglone or KPT6566. **(A)** Flow cytometric cell sorting of parental Caco-2 cells (top panel), CD44^+^CD133^+^ Caco-2 cells (middle panel), and ΔCD44^+^CD133^+^ Caco-2 cells (bottom panel) double-labeled with anti-CD44 and anti-CD133 antibodies. Sorted tumor-initiating Caco-2 cells (CD44^+^CD133^+^ subpopulation) and non-tumor-initiating Caco-2 cells (ΔCD44^+^CD133^+^ subpopulation) were re-analyzed immediately following the first sorting. The percentages refer to the purity of the selected subpopulation contained in the sorted fraction. **(B)** Expression of Pin1 mRNA by the CD44^+^CD133^+^ and ΔCD44^+^CD133^+^ subpopulations of Caco-2 cells. Analysis of the expression level of Pin1 mRNA was performed in CD44^+^CD133^+^ or ΔCD44^+^CD133^+^ subpopulations of Caco-2 cells using RT-PCR. Following RT-PCR, an aliquot of each amplified sample was visualized by agarose gel electrophoresis and ethidium bromide staining. β-actin was used as a reference gene for normalization. **(C)** Quantitative real-time PCR analyses of Pin1 mRNA expression. Quantitative analysis of Pin1 mRNA was performed in CD44^+^CD133^+^ or ΔCD44^+^CD133^+^ subpopulations of Caco-2 cells using real-time PCR. Expression of Pin1 by the ΔCD44^+^CD133^+^ subpopulation of Caco-2 cells was set at 100%. Data are presented as the mean ± S.D. β-actin was used as a control for normalization. **(D)** Expression of Pin1 protein. Western blot analysis of total cell extracts prepared from the CD44^+^CD133^+^ and ΔCD44^+^CD133^+^ subpopulations of Caco-2 cells. Aliquots were separated by SDS-PAGE (15% gels for Pin1 and 12% gels for α/β-tubulin). The proteins were immunoblotted using anti-Pin1 or anti-α/β-Tubulin antibodies. The positions of the molecular mass marker proteins (New England Biolabs) are shown on the left of the gel (molecular mass in kDa). Similar results were obtained from three independent experiments. **(E)** Suppression of colony formation by CD44^+^CD133^+^ and ΔCD44^+^CD133^+^ Caco-2 cells by Juglone. The CD44^+^CD133^+^ and ΔCD44^+^CD133^+^ subpopulations of Caco-2 cells were seeded at a density of 2.5 × 10^3^ per well and cultured in medium containing 0, 0.625, 1.25, 2.5, 5, or 10 μM Juglone. Five days later, colonies were stained with 0.05% (w/v) Crystal Violet. Results are representative of three independent experiments, each with similar results. **(F)** Relative colony numbers after Juglone treatment. Colonies of CD44^+^CD133^+^ and ΔCD44^+^CD133^+^ Caco-2 cells were counted after staining with Crystal Violet, and the number of colonies formed was compared with that formed by the untreated groups (DMSO) of CD44^+^CD133^+^ or ΔCD44^+^CD133^+^ Caco-2 cells. The value for the control group was normalized to 100. Values represent the mean ± S.D. An unpaired Student’s t-test was used to test statistical significance. **p < 0.01, versus control cells. Three independent experiments yielded similar results **(G)** Morphology of CD44^+^CD133^+^ and ΔCD44^+^CD133^+^ Caco-2 cells exposed to Juglone. CD44^+^CD133^+^ and ΔCD44^+^CD133^+^ Caco-2 cells (1 × 10^4^) were incubated with different doses of Juglone and monitored at 6 days under an inverted phase-contrast microscope (IX71; Olympus, Tokyo, Japan). Scale bars, 100 μm. Three independent experiments yielded similar results. **(H)** Inhibition of CD44^+^CD133^+^ and ΔCD44^+^CD133^+^ Caco-2 cell proliferation by Juglone. CD44^+^CD133^+^ and ΔCD44^+^CD133^+^ Caco-2 cells were treated with indicated concentrations of Juglone, and the number of cells was counted using a hemocytometer. Inhibition of cell growth is presented as a percentage of the untreated control (DMSO) cell value at a given concentration of Juglone. Data are presented as the mean ± S.D. of three independent experiments. The sigmoidal dose-response curve provided by SoftMax Pro software was used to calculate the IC_50_ value for Juglone. Three independent experiments were performed, all of which gave similar results. **(I)** Suppression of colony formation by the CD44^+^CD133^+^ and ΔCD44^+^CD133^+^ subpopulations of Caco-2 cells by KPT6566. The CD44^+^CD133^+^ and ΔCD44^+^CD133^+^ subpopulations of Caco-2 cells were seeded at a density of 2.5 × 10^3^ per well and cultured in medium containing 0, 1.25, 2.5, 5, 10, or 20 μM KPT6566. Five days later, colonies were stained with 0.05% (w/v) Crystal Violet. Three independent experiments were performed, and all yielded similar results. **(J)** Relative colony numbers after KPT6566 treatment. Colonies of CD44^+^CD133^+^ and ΔCD44^+^CD133^+^ Caco-2 cells were counted after Crystal Violet staining, and the number of colonies formed was compared with that formed by the untreated groups (DMSO) of CD44^+^CD133^+^ or ΔCD44^+^CD133^+^ Caco-2 cells. The value for the control group was normalized to 100. Values represent the mean ± S.D. An unpaired Student’s t-test was used to test statistical significance. **p < 0.01, versus control cells. Three independent experiments yielded similar results. **(K)** Morphology of CD44^+^CD133^+^ and ΔCD44^+^CD133^+^ Caco-2 cells exposed to KPT6566. CD44^+^CD133^+^ and ΔCD44^+^CD133^+^ Caco-2 cells (1 × 10^4^) were incubated with different doses of KPT6566 and monitored at 6 days under an inverted phase-contrast microscope (IX71; Olympus, Tokyo, Japan). Scale bars, 100 μm. Three independent experiments yielded similar results. **(L)** Inhibition of CD44^+^CD133^+^ and ΔCD44^+^CD133^+^ Caco-2 cell proliferation by KPT6566. CD44^+^CD133^+^ and ΔCD44^+^CD133^+^ Caco-2 cells were treated with the indicated concentrations of KPT6566, and the number of cells was counted using a hemocytometer. Inhibition of cell growth is presented as a percentage of the untreated control (DMSO) cell number at a given concentration of KPT6566. Data are described as the mean ± S.D. of three independent experiments. The sigmoidal dose-response curve provided by SoftMax Pro software was used to calculate the IC_50_ value for KPT6566. Three independent experiments were performed, all of which gave similar results.

Recent studies suggest that tumor-initiating cells are enriched after chemotherapy; this is because tumor-initiating cells can resist and proliferate despite the fact that many classic chemotherapeutic drugs kill the bulk of the tumor (Dean et al., 2005; Tredan et al., 2007; Phi et al., 2018; Li et al., 2021). Based on these observations, we postulated that tumor-initiating Caco-2 cells might be more resistant to Juglone or KPT6566 than non-tumor-initiating Caco-2 cells. To investigate whether Juglone or KPT6566 can eliminate tumor-initiating cells in CRC, Caco-2 cells were divided into a tumor-initiating subpopulation (CD44^+^CD133^+^-positive Caco-2 cells) and a non-tumor-initiating subpopulation (CD44^+^CD133^+^-negative Caco-2 cells; tumor-initiating cell-depleted Caco-2 cells). As we reported recently (Kim et al., 2021), unsorted parental Caco-2 cells comprised three representative subpopulations (non-tumor-initiating CD44^−^CD133^-^ cells, non-tumor-initiating CD44^−^CD133^+^ cells, and tumor-initiating CD44^+^CD133^+^ cells); however, a CD44^+^CD133^−^ Caco-2 fraction was barely detectable (Figure 5A top panel). To evaluate whether the CD44^+^CD133^+^ subpopulation of tumor-initiating Caco-2 cells was isolated successfully by FACS, we monitored these cells by flow cytometry analysis immediately after the primary sorting. In the same way, the ΔCD44^+^CD133^+^ non-tumor-initiating cell population was also assessed to evaluate if the CD44^+^CD133^+^ tumor-initiating Caco-2 cells were eliminated successfully. As shown in Figure 5A, the fraction of CD44^+^CD133^+^ tumor-initiating Caco-2 cells in the sorted CD44^+^CD133^+^ subpopulation was about 87% (middle panel), and less than 2% of the isolated ΔCD44^+^CD133^+^ subpopulation was tumor-initiating CD44^+^CD133^+^ cells (bottom panel), indicating that both populations of Caco-2 cells were isolated successfully.

Despite the overexpression of Pin1 by Caco-2 cells as demonstrated in Figure 1A, it is unclear whether CD44^+^CD133^+^ tumor-initiating Caco-2 cells express Pin1. To confirm this, total RNA from the CD44^+^CD133^+^ and ΔCD44^+^CD133^+^ subpopulations of Caco-2 cells were purified, and RT-PCR was performed. PCR products derived from Pin1 mRNA were observed in tumor-initiating Caco-2 cells and in non-tumor-initiating Caco-2 cells (Figure 5B). We sequenced these RT-PCR products and confirmed them to represent the reported human Pin1 cDNA sequence (data not shown). These results demonstrate the existence of Pin1 mRNA in the CD44^+^CD133^+^ tumor-initiating cell subpopulation of Caco-2 cells.

We were also able to quantify Pin1 mRNA levels using quantitative real-time PCR. Interestingly, human Pin1 mRNA was about 2-fold more abundant in the CD44^+^CD133^+^ subpopulation than in the ΔCD44^+^CD133^+^ subpopulation (Figure 5C).

The amounts of Pin1 protein in the CD44^+^CD133^+^ and ΔCD44^+^CD133^+^ subpopulations of Caco-2 cells were determined by western blotting. As shown in Figure 5D, the Pin1 protein was expressed at similar or slightly higher levels in the CD44^+^CD133^+^ subpopulation than in the ΔCD44^+^CD133^+^ subpopulation.

To investigate whether Juglone affects the colony forming potential of CD44^+^CD133^+^ tumor-initiating Caco-2 cells, FACS-isolated CD44^+^CD133^+^ tumor-initiating Caco-2 cells and ΔCD44^+^CD133^+^ non-tumor-initiating Caco-2 cells were treated with different amounts of Juglone. Final measurements were made 5 days after treatment. When comparing the inhibitory effect of Juglone on the colony formation ability of CD44^+^CD133^+^ cells and ΔCD44^+^CD133^+^ Caco-2 cells, we noticed that Juglone repressed the colony forming ability of CD44^+^CD133^+^ and ΔCD44^+^CD133^+^ cells in a concentration-dependent manner (Figure 5E). A colony formation assay of both subpopulations showed that the number of colonies was significantly lower after Juglone treatment (Figure 5F), indicating that Juglone might be an effective inhibitor of CD44^+^CD133^+^ tumor-initiating Caco-2 cell-mediated tumorigenesis.

The growth potential of CD44^+^CD133^+^ and ΔCD44^+^CD133^+^ cells after Juglone treatment was also monitored by phase-contrast microscopy. Juglone meaningfully suppressed the growth of both CD44^+^CD133^+^ tumor-initiating Caco-2 cells and ΔCD44^+^CD133^+^ non-tumor-initiating Caco-2 cells in a concentration-dependent manner (Figure 5G). Interestingly, the CD44^+^CD133^+^ and ΔCD44^+^CD133^+^ subpopulations of Caco-2 cells displayed similar sensitivity to Juglone, although tumor-initiating cells generally show resistance, or lower sensitivity, to chemotherapeutic drugs (Phi et al., 2018; Li et al., 2021). The IC_50_ values of Juglone for inhibiting cell growth were 1.38 μM for CD44^+^CD133^+^ tumor-initiating Caco-2 cells and 1.21 μM for ΔCD44^+^CD133^+^ non-tumor-initiating Caco-2 cells (Figure 5H), suggesting Juglone is a small-molecule compound targeting CD44^+^CD133^+^ cells to inhibit their tumorigenic tendency.

The anti-tumorigenic potential of KPT6566 was also analyzed in a clonogenic assay and by growth curve analysis of CD44^+^CD133^+^ tumor-initiating Caco-2 cells. Interestingly, KPT6566 also significantly affected colony formation by CD44^+^CD133^+^ Caco-2 cells. As presented in Figure 5I, KPT6566 inhibited colony formation by the CD44^+^CD133^+^ and ΔCD44^+^CD133^+^ subpopulations of Caco-2 cells in a concentration-dependent manner. Quantification of the colony formation assays for both groups showed that the number of colonies was significantly lower after KPT6566 treatment (Figure 5J), indicating that KPT6566 might also be an effective inhibitor of CD44^+^CD133^+^ tumor-initiating Caco-2 cells.

The proliferative properties of CD44^+^CD133^+^ cells and ΔCD44^+^CD133^+^ cells after KPT6566 treatment were monitored by microscopy. KPT6566 also suppressed the growth of the CD44^+^CD133^+^ and ΔCD44^+^CD133^+^ subpopulations in a concentration-dependent manner (Figure 5K). Similar to Juglone in Figure 5H, the CD44^+^CD133^+^ and ΔCD44^+^CD133^+^ subpopulations of Caco-2 cells showed similar sensitivity to KPT6566. The IC_50_ values of KPT6566 for inhibiting cell growth were 5.76 μM for CD44^+^CD133^+^ tumor-initiating Caco-2 cells and 5.39 μM for ΔCD44^+^CD133^+^ non-tumor-initiating Caco-2 cells (Figure 5L), suggesting KPT6566 as another small-molecule compound targeting CD44^+^CD133^+^ cells. Taken together, these data suggest that targeting the Pin1 protein might be a preferential and effective strategy for killing tumor-initiating CRC cells.

One of the important characteristics of tumor-initiating cells is high expression levels of ATP-binding cassette (ABC) transporters (Phi et al., 2018; Li et al., 2021), therefore, we performed RT-PCR to detect expression of representative multidrug resistance genes. As shown in Supplementary Figure S2, mRNAs encoding ABCG2 and ABCB1 were more abundant in CD44^+^CD133^+^ tumor-initiating Caco-2 cells than in ΔCD44^+^CD133^+^ non-tumor-initiating Caco-2 cells (i.e., the CD44^−^CD133^-^ and CD44^−^CD133^+^ fractions). However, the expression levels of ABCC1 and ABCC2 drug efflux transporters were very similar between the non-tumor-initiating subpopulation (CD44^−^CD133^-^ and CD44^−^CD133^+^ Caco-2 cells) and the tumor-initiating subpopulation (CD44^+^CD133^+^ Caco-2 cells).

### Juglone and KPT6566 Reduce CD44^+^CD133^+^ Tumor-Initiating Caco-2 Cell-Mediated Tumor Formation in NSG Mice

**FIGURE 6:**
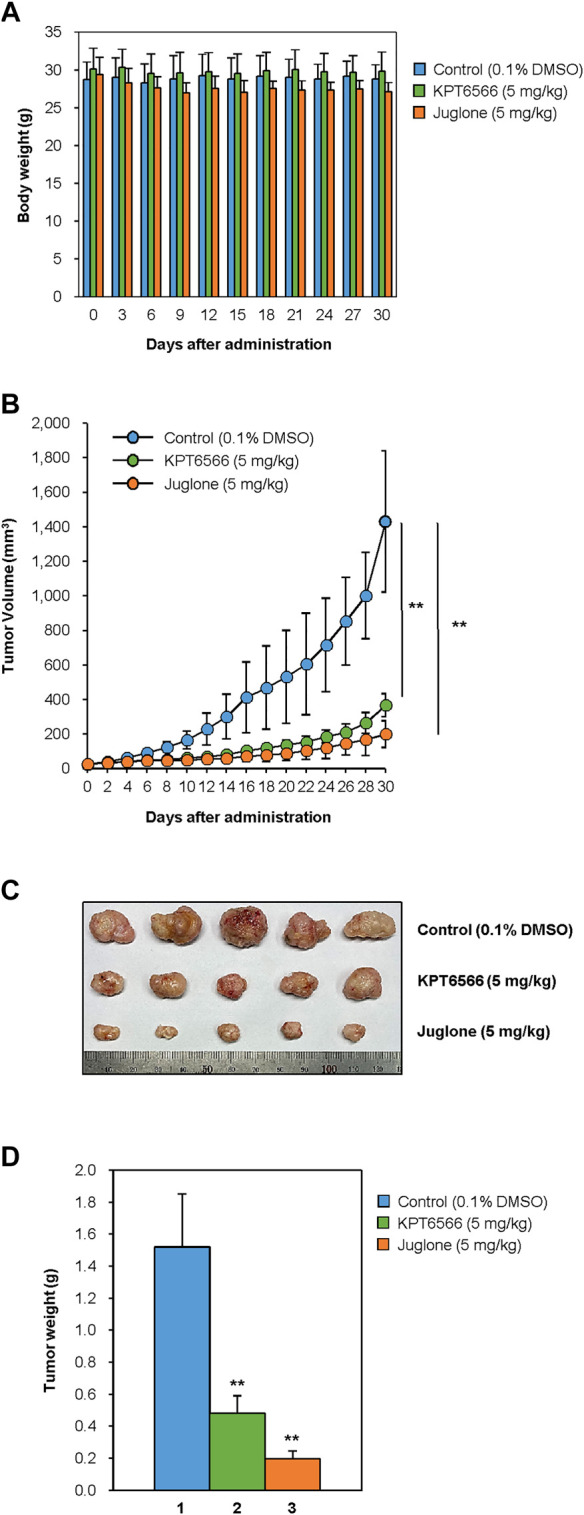
Suppression of CD44^+^CD133^+^ tumor-initiating Caco-2 cell-induced tumor formation by Juglone and KPT6566. **(A)** Average weight of NSG mice after treatment with Juglone or KPT6566. Body weight was checked at 3 days intervals to evaluate the potential toxicity of Juglone or KPT6566 throughout the experiment. No significant weight loss was observed. **(B)** Reduction in tumor volume after injection of Juglone or KPT6566. Tumor-bearing mice were treated by intraperitoneal injection of vehicle, Juglone (5 mg/kg), or KPT6566 (5 mg/kg) at 3 days intervals for 30 days. Tumor size was measured at 2 days intervals throughout the experiment. Values are expressed as the mean ± S.D., and statistical analysis was assessed using an unpaired Student’s t-test. **p < 0.01, versus control cells. **(C)** Photograph and comparison of excised tumors. Tumors were removed from mice treated with vehicle control, Juglone, or KPT6566. Photographs were taken 30 days after Juglone or KPT6566 treatment. **(D)** Quantification of tumor size after Juglone or KPT6566 treatment. Weight of tumors from control, Juglone-, or KPT6566-treated mice were plotted. Data are expressed as the mean ± S.D. Statistical analysis was done using an unpaired Student’s t-test. **p < 0.01, versus control cells.

Finally, to investigate the effects of Juglone or KPT6566 treatment on tumor-initiating cell-induced tumorigenesis, the *in vivo* anti-tumorigenic properties of Juglone and KPT6566 were examined in NSG mice bearing CD44^+^CD133^+^ Caco-2 cell xenografts. Approximately 1 × 10^5^ CD44^+^CD133^+^ tumor-initiating Caco-2 cells were suspended in 100 μl of injection solution and inoculated subcutaneously in the flanks of NSG mice. When tumors had grown to 15–25 mm^3^, the mice were arbitrarily separated into three groups and treated with Juglone (5 mg/kg), KPT6566 (5 mg/kg), or vehicle (0.1% DMSO) once every 3 days for 30 days. We first evaluated the adverse effects of Juglone or KPT6566 in tumor-bearing NSG mice. To check the health status of mice treated with Juglone or KPT6566, mean body weight of mice was measured at 3 days intervals throughout the experimental period. The body weight of NSG mice treated with Juglone or KPT6566 was similar to that of vehicle-treated NSG mice. These results suggest that Juglone or KPT6566 themselves had no significant toxic effects in these experiments (Figure 6A).

Next, we investigated the antitumor effects of Juglone or KPT6566. Consistent with the cell-based experiments (Figure 5), tumors in mice treated with Juglone or KPT6566 were significantly smaller than those in control mice (Figure 6B). These results indicate that Pin1 is critical for CD44^+^CD133^+^ tumor-initiating cell-mediated tumorigenesis, and that suppression of Pin1 function significantly impairs tumor growth *in vivo*. Compared with the vehicle group (1431.1 ± 409.4 mm^3^) at the end of treatment, Juglone (199.7 ± 67.1 mm^3^) or KPT6566 (367.0 ± 76.9 mm^3^) reduced tumor volume by 86.0% (*n* = 5, *p* < 0.01) and 74.4% (*n* = 5, *p* < 0.01), respectively.

The weight of the tumors in mice treated intraperitoneally with Juglone or KPT6566 was much smaller than that of tumors in the vehicle-treated group (Figure 6C). Although the average tumor weight in the control group was 1.52 ± 0.33 g, that in the Juglone-treated group was 0.20 ± 0.05 g, and that in the KPT6566-treated group was 0.48 ± 0.11 g at 30 days after treatment (Figure 6D). Compared with the control group at the end of treatment, Juglone or KPT6566 treatment decreased tumor weight by 86.8% (*p* < 0.01) and 68.4% (*p* < 0.01), respectively. Taken together, these results suggest that Pin1 activity is essential for CD44^+^CD133^+^ tumor-initiating Caco-2 cell-induced tumorigenesis *in vivo*, and that Juglone or KPT6566 effectively inhibit tumor-initiating Caco-2 cell-mediated tumor growth.

## Discussion

Our current data show that two potent chemical Pin1 inhibitors, Juglone and KPT6566, induce apoptotic cell death (Figure 4) and inhibit the tumorigenicity of CD44^+^CD133^+^ tumor-initiating Caco-2 CRC cells (Figure 6). CRC develops from the epithelial cells of the lining of colon or rectum. It is known that a fraction of CRC cells within a tumor harbor have high tumorigenic potential; these cells are called tumor-initiating cells. Although they account for only a subpopulation of cells, tumor-initiating cells show stem or progenitor cell-like characteristics and have a unique ability for asymmetric cell division (Bu et al., 2013; Gupta et al., 2019; Kim et al., 2021). The results of our previous study show that Wnt/β-catenin signaling is over-active in tumor-initiating Caco-2 cells (Kim et al., 2021). Consistent with these results, other studies show that CRC can become advanced due to dysregulation of the Wnt/β-catenin signaling pathway (Zhou et al., 2018; Xie et al., 2020). Despite rapid progression in surgery, chemo-, radio-, and immune therapies, CRC remains one of the deadliest human diseases worldwide. In particular, colorectal tumor-initiating cells are responsible for chemo- and radiotherapy resistance and pose a high risk of tumor relapse. Indeed, conventional chemotherapeutic drugs and radiotherapy may kill the bulk of the tumor, but not kill tumor-initiating cells (Tredan et al., 2007; Visvader and Lindeman, 2008; Li et al., 2021). Thus, a better understanding of the cancerous properties of tumor-initiating colorectal cancer cells is required for selective treatment of malignant CRC.

Our data show that Juglone and KPT6566 suppress CRC cell proliferation and colony formation (Figure 1). These findings prompted us to identify the molecular mechanisms underlying inhibition of cell growth and colony formation by CRC cells. In general, human cancers are characterized by uncontrolled and unlimited cell growth (Hanahan and Weinberg, 2011) caused by aberrant activation or inactivation of various cell cycle regulator proteins (Otto and Sicinski, 2017). Many positive cell cycle regulatory proteins, such as cyclins and CDKs, are overexpressed, whereas several CDK inhibitors are downregulated. In addition, perturbation of cell signaling pathways is common. The fact that treatment with Juglone or KPT6566 inhibited expression of β-catenin and cyclin D1 in Caco-2 cells (Figure 3) further supports the conclusion that overexpression of Pin1 and β-catenin is closely related to development of human CRC (Kim et al., 2005), and that Pin1 plays an essential role in colorectal carcinogenesis by regulating cyclin D1 expression (Kuramochi et al., 2006). In addition, Pin1 overexpression might be attributable to stabilization of β-catenin and cyclin D1 in human colorectal tumor-initiating cells.

According to the current study, the effect of Pin1 inhibition is not limited to downregulation of β-catenin and cyclin D1 in Caco-2 cells. We also demonstrated that suppression of Pin1 by Juglone or KPT6566 downregulates cyclin D2 and cyclin D3 in Caco-2 cells (Figure 3). Additionally, inactivation of Pin1 also led to a reduction in CDK4 and CDK6 protein levels in Caco-2 cells (Figure 3), suggesting that some biological effects of Juglone or KPT6566 could also be mediated by downregulation of CDK4 and CDK6. D-type cyclin proteins comprise three subtypes (cyclin D1, cyclin D2, and cyclin D3) which collectively control early G1-phase progression by modulating the activity of CDK4 and CDK6 (Sherr, 2000). Although these results support the concept that Pin1 promotes cancer progression, and that Juglone and KPT6566 suppress the tumorigenic potential of colorectal cancer, it remains unclear whether Pin1 inhibitors have similar effects on other solid tumors. Overall, our results suggest that Juglone and KPT6566 suppress proliferation of Caco-2 cells by downregulating expression cyclin D1, cyclin D2, cyclin D3, CDK4, and CDK6, and that this may contribute to the anti-tumorigenic effects of Juglone or KPT6566 against CD44^+^CD133^+^ tumor-initiating Caco-2 cells. Taken together, the results show that Pin1 plays an essential role in human CRC development by regulating D-type cyclins and D-type-cyclin-dependent kinases.

Pin1 also plays a crucial role in tumorigenesis mediated by CD44^+^CD133^+^ tumor-initiating Caco-2 cells. Although studies show that high Pin1 expression is associated with progression of human CRC (Kim et al., 2005; Kuramochi et al., 2006), no study has examined the effect of the Pin1 protein on the tumorigenicity of tumor-initiating colorectal cancer cells. Here, we showed that two chemical inhibitors of Pin1, Juglone and KPT6566, inhibit growth of colorectal tumor-initiating cells and colony formation (Figure 5). Furthermore, Juglone and KPT6566 suppress the tumorigenic properties of CD44^+^CD133^+^ tumor-initiating Caco-2 cells (Figure 6), demonstrating a critical role for Pin1 in promoting the tumorigenic potential of these cells. However, although the present data suggest that targeting Pin1 in CD44^+^CD133^+^ colorectal tumor-initiating cells is a promising therapeutic approach, there are some limitations regarding the use of Pin1 inhibitors for treating human disease. Pin1 plays a pivotal role in protection against age-dependent neurodegeneration, and an essential role in preventing tau-related and Aβ pathologies in Alzheimer’s disease (Liou et al., 2003; Lee et al., 2011; Wang et al., 2020). Therefore, additional studies are required to investigate comprehensive Pin1 functions in tumor-initiating cells and during age-dependent neurodegeneration.

Despite the noteworthy achievements in handling human cancers, acquired drug resistance remains a significant problem when treating cancer. In past decades, chemoresistance has led to the failure of therapeutic drug treatments, which is a major limitation of current cancer therapy (Tredan et al., 2007; Van Der Jeught et al., 2018). Accumulating evidence suggests that tumor-initiating cells are responsible for drug resistance and cancer relapse (Zhou et al., 2018; Li et al., 2021). However, chemical inhibitors of Pin1 might be potential therapeutic agents that target both tumor-initiating and non-tumor-initiating cells. According to our results, the IC_50_ of Juglone and KPT6566 for CD44^+^CD133^+^ tumor-initiating Caco-2 cells and ∆CD44^+^CD133^+^ non-tumor-initiating Caco-2 cells was similar (Figure 5). Conventional chemotherapy focuses mainly on eradicating the bulk cancer; however, most therapies are ineffective against tumor-initiating cells. In this respect, targeting Pin1 could offer a breakthrough. Overexpression of ABC membrane transporters by tumor-initiating cells leads to enhanced multidrug efflux capability and a reduction in the drug dose delivered at a cellular level, resulting in efflux-mediated drug resistance and cytotoxic drug treatment failure (Dean et al., 2005; Visvader and Lindeman, 2008; Alison et al., 2011; Li et al., 2021). Though the molecular mechanisms underlying Pin1 function in colorectal tumor-initiating cells require further study, it is not surprising that targeting Pin1 is considered a novel strategy to eliminate tumor-initiating cells and reduce the risk of cancer recurrence.

In summary, we show here that Juglone and KPT6566 inhibit the tumorigenic potential of CD44^+^CD133^+^ tumor-initiating Caco-2 cells. Although chemical Pin1 inhibitors elicit potent antitumor effects against several human cancers, their inhibitory impact on the tumorigenic capacity of CD44^+^CD133^+^ tumor-initiating Caco-2 cells was unknown. We show that Pin1 plays a central role in promoting the tumorigenic potential of CD44^+^CD133^+^ tumor-initiating Caco-2 cells. We also show that Juglone and KPT6566 suppress CRC cell growth and colony formation and inhibit the tumorigenic properties of CD44^+^CD133^+^ tumor-initiating Caco-2 cells. Thus, targeting Pin1 in CD44^+^CD133^+^ tumor-initiating cells is a promising therapeutic approach to treating human CRC.

## Data Availability

The original contributions presented in the study are included in the article/Supplementary Material, further inquiries can be directed to the corresponding author.
