# Supplementary material for: Juglone and KPT6566 Suppress the Tumorigenic Potential of CD44+CD133+ Tumor-Initiating Caco-2 Cells In Vitro and In Vivo
Source: Front Cell Dev Biol. 2022 Mar 30;10:861045. doi: 10.3389/fcell.2022.861045 (PMC9006057; doi:10.3389/fcell.2022.861045)
Supplement: Supplementary file 1 [file DataSheet1.PDF]

Juglone and KPT6566 Suppress the Tumorigenic Potential of $CD44^{+}CD133^{+}$ Tumor-Initiating Caco-2 Cells *in vitro* and *in vivo*

Junghoon Kim, Seonock Lee, Ruijing Sun, and Jungho Kim

Supplementary Material

Supplementary Materials and Methods

Immunocytochemical analysis

Human CRC cells grown on a cell culture slide (SPL Lifer Sciences) were fixed for 10 min at room temperature with 4% paraformaldehyde and immunocytochemical analysis was performed as previously described (Smalheiser and Kim, 1995;Hisa et al., 2011). To detect Pin1, slides were incubated overnight at 4°C with an anti-Pin1 antibody (Proteintech) in blocking buffer, followed by an HRP-conjugated anti-rabbit antibody (GBI Labs). After mounting the cells in a drop of Acrymount Plus mounting medium (StatLab), images were viewed under an inverted phase-contrast microscope (IX71, Olympus).

RT-PCR for ATP binding cassette transporter genes

RT-PCR to amplify *ABCG2, ABCB1, ABCC1,* and *ABCC2* was performed in triplicate. β-actin mRNA was used as an internal control. The following primers were used: ABCG2, 5'-GATAAAGTGGCAGACTCCAAGGT-3' and 5'-CCAATAAGGTGAGGCTATCAAACA-3'; ABCB1, 5'-GCTCCTGACTATGCCAAAGC-3' and 5'-TCTTCACCTCCAGGCTCAGT-3'; ABCC1, 5'-AGGTGGACCTGTTTCGTGAC-3' and 5'-ACCCTGTGATCCACCAGAAG-3', and ABCC2, 5'-AGGTTTGCCAGTTATCCGTG-3' and 5'-AACAAAGCCAACAGTGTCCC-3'.

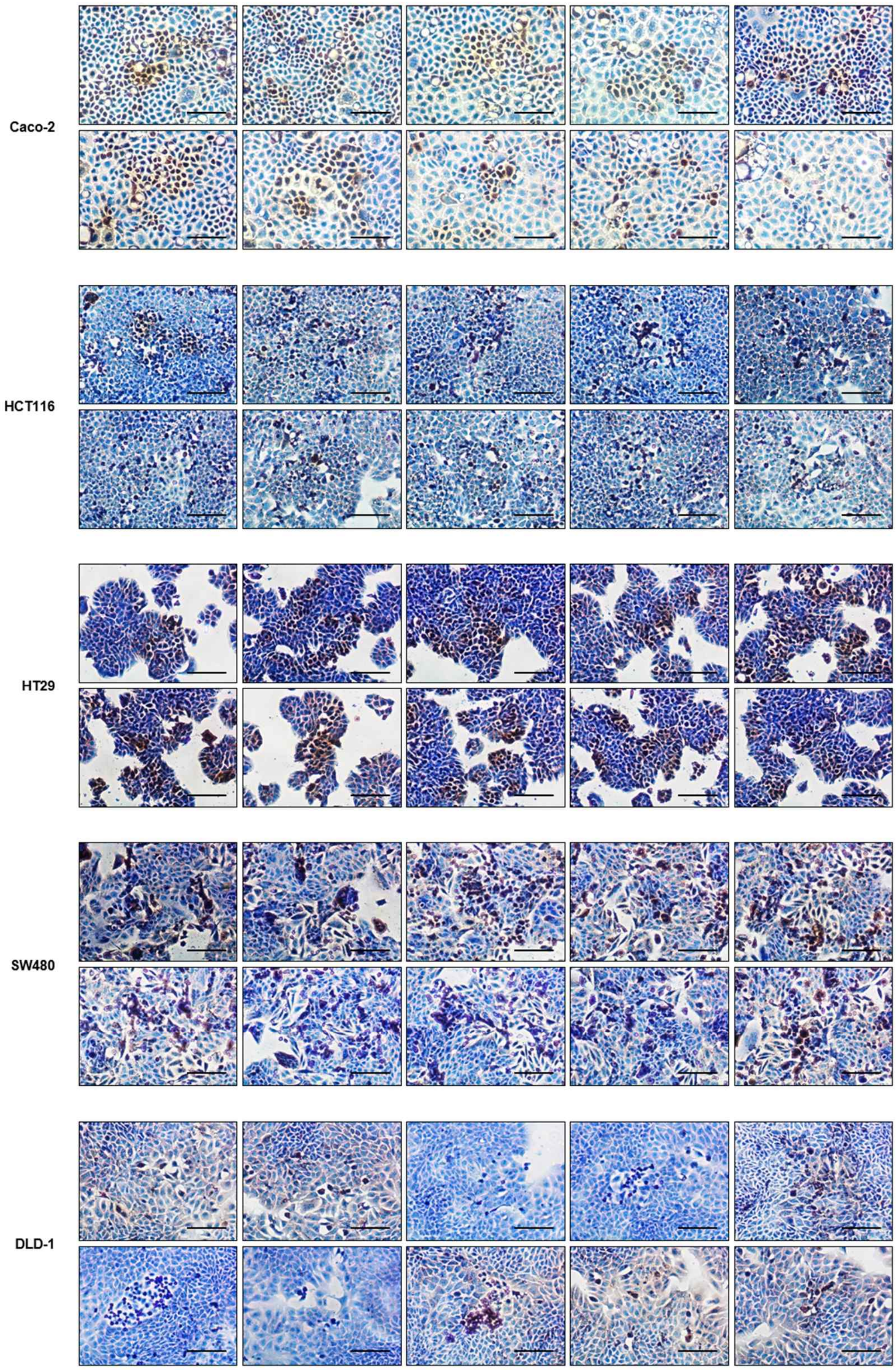

Supplementary Figure S1. Immunocytochemical expression of Pin1 in human CRC cell lines. The immunocytochemical investigation revealed a focal pattern of Pin1 expression in Caco-2 (top panels), HCT116 (second panels), HT29 (third panels), SW480 (fourth panels), and DLD-1 (bottom panels) cells. Images were taken under an inverted phase-contrast microscope (IX71, Olympus).

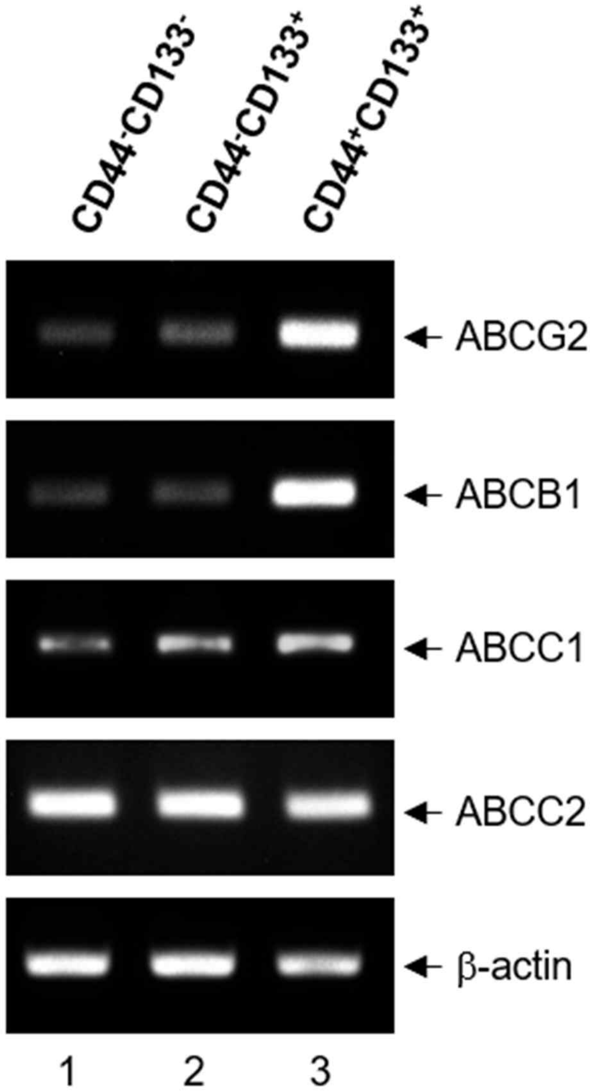

Supplementary Figure S2. Expression of ATP-binding cassette (ABC) transporter mRNAs by the $CD44^{-}CD133^{-}$, $CD44^{-}CD133^{+}$, and $CD44^{+}CD133^{+}$ subpopulations of Caco-2 cells. Analysis of ABCG2, ABCB1, ABCC1, and ABCC2 mRNA expression by the $CD44^{-}CD133^{-}$, $CD44^{-}CD133^{+}$, or $CD44^{+}CD133^{+}$ subpopulations of Caco-2 cells by RT-PCR. Following RT-PCR, an aliquot of each amplified sample was visualized by agarose gel electrophoresis and ethidium bromide staining. β-actin was used as a reference gene for normalization.

Supplementary Reference

Hisa, I., Inoue, Y., Hendy, G.N., Canaff, L., Kitazawa, R., Kitazawa, S., Komori, T., Sugimoto, T., Seino, S., and Kaji, H. (2011). Parathyroid hormone-responsive Smad3-related factor, Tmem119, promotes osteoblast differentiation and interacts with the bone morphogenetic protein-Runx2 pathway. *J Biol Chem* 286**,** 9787-9796.

Smalheiser, N.R., and Kim, E. (1995). Purification of cranin, a laminin binding membrane protein. Identity with dystroglycan and reassessment of its carbohydrate moieties. *J Biol Chem* 270**,** 15425-15433.
